# Maternal and neonatal risk factors for childhood type 1 diabetes: a matched case-control study

**DOI:** 10.1186/1471-2458-10-281

**Published:** 2010-05-27

**Authors:** Lynn Robertson, Kirsten Harrild

**Affiliations:** 1 NHS Grampian, Summerfield House, 2 Eday Road, Aberdeen, AB15 6RE, UK; 2 University of Aberdeen, Medical Statistics Team, Section of Population Health, Polwarth Building, Foresterhill, Aberdeen, AB25 2ZD, UK

## Abstract

**Background:**

An interaction between genetic susceptibility and environmental factors is thought to be involved in the aetiology of type 1 diabetes. The aim of this study was to investigate maternal and neonatal risk factors for type 1 diabetes in children under 15 years old in Grampian, Scotland.

**Methods:**

A matched case-control study was conducted by record linkage. Cases (n = 361) were children born in Aberdeen Maternity Hospital from 1972 to 2002, inclusive, who developed type 1 diabetes, identified from the Scottish Study Group for the Care of Diabetes in the Young Register. Controls (n = 1083) were randomly selected from the Aberdeen Maternity Neonatal Databank, matched by year of birth. Exposure data were obtained from the Aberdeen Maternity Neonatal Databank. Conditional logistic regression was used to evaluate the association between various maternal and neonatal factors and the risk of type 1 diabetes.

**Results:**

There was no evidence of statistically significant associations between type 1 diabetes and maternal age, maternal body mass index, previous abortions, pre-eclampsia, amniocentesis, maternal deprivation, use of syntocinon, mode of delivery, antepartum haemorrhage, baby's sex, gestational age at birth, birth order, birth weight, jaundice, phototherapy, breast feeding, admission to neonatal unit and Apgar score (*P *> 0.05). A significantly decreased risk of type 1 diabetes was observed in children whose mothers smoked at the booking appointment compared to those whose mothers did not, with an adjusted OR of 0.67, 95% CI (0.46, 0.99).

**Conclusions:**

This case-control study found limited evidence of a reduced risk of the development of type 1 diabetes in children whose mothers smoked, compared to children whose mothers did not. No evidence was found of a significant association between other maternal and neonatal factors and childhood type 1 diabetes.

## Background

It has recently been reported that if present trends continue, new cases of type 1 diabetes in European children younger than 5 years will double between 2005 and 2020, and prevalent cases younger than 15 years will rise by 70% [1]. In Scotland, between 1984 and 2003 the incidence of childhood type 1 diabetes increased by 2.6% per year, and this rise has been greater in children aged under 5 years [2]. In 2007, there were approximately 27,000 people with type 1 diabetes in Scotland, of which about 1,800 were under 15 years of age [3].

An interaction between genetic susceptibility and environmental factors is thought to be involved in the aetiology of type 1 diabetes [4]. Many investigators have focussed on the relationship between maternal and neonatal factors and the subsequent risk of type 1 diabetes. However, the evidence on the role of many maternal and neonatal factors in the development of childhood type 1 diabetes is inconclusive and only one study has presented data on the Scottish population [5]. Several studies have reported a significant increased risk of type 1 diabetes with increasing maternal age [6-8], while others have reported no association [9,10]. A recently published meta-analysis[11] reported a weak, but significant, increase in the risk of type 1 diabetes with increasing maternal age. Another meta-analysis demonstrated an increase in the risk of childhood type 1 diabetes after Caesarean section delivery [12]. A further recent meta-analysis has reported a relatively small, but significant, increase in risk in children who are heavier at birth[13]. Some studies have reported a decreased risk with increasing birth order [6,8], others have found no association [9,14].

The aim of this study was to investigate the relationship between several maternal and neonatal factors, including maternal age, and the risk of type 1 diabetes in children under 15 years in Grampian, Scotland.

## Methods

A matched case-control study was carried out, based on the linkage between two databases, the Scottish Study Group for the Care of Diabetes in the Young (SSG) Register and the Aberdeen Maternity Neonatal Databank (AMND). The SSG Register started collecting data from January 1985 on all children diagnosed with diabetes under 15 years in Scotland [15]. All diabetes centres in Scotland are requested to report to this register by providing details of new patients diagnosed with type 1 diabetes. The AMND has prospectively recorded information on all births and pregnancy related events occurring in Aberdeen city and districts since 1950 [16].

Cases were defined as children diagnosed with type 1 diabetes under 15 years in the Grampian region from 1984 to 2005 inclusive, who were born in Aberdeen Maternity Hospital (AMH), and were identified by linking the AMND with the SSG Register. The SSG Register Officer provided data on children diagnosed in the Grampian region from 1984 to 2005 inclusive, which included their Community Health Index (CHI) number, name, sex and date of birth. Using CHI numbers, data were linked to the hospital patient administration system to extract hospital unit numbers. Hospital unit numbers were then used to link to babies on the AMND. Those records that were not successfully linked were for children who were not born at AMH and were therefore ineligible for this study. Anonymised birth data were extracted for all children with type 1 diabetes linked with their birth records. Birth data were also extracted for all other children born during the same years as children with type 1 diabetes. With the exception of multiple births, which were excluded because of the difficulties in distinguishing between the details of the neonates, all identified cases were included in the study. For each case, three controls matched on the year of birth were randomly selected from the same population. This study used data collected over 30 years, during which time there may have been changes in environmental exposures, including pregnancy and childbirth behaviours and practices or changes in obstetric protocols. There has also been an increase in the incidence of type 1 diabetes over this time[1]. For this reason, the controls were matched on the year of birth to take account of confounding by factors that may have changed over the period of study.

Information relating to the following variables were collected from the AMND: maternal type 1 diabetes, maternal and paternal age, maternal body mass index (BMI) and maternal smoking (recorded at the first booking appointment by approximately 16 weeks gestation), previous abortions, pre-eclampsia, amniocentesis, maternal viral infection, maternal bacterial disease, maternal deprivation (Carstairs and Morris [17]), syntocinon, mode of delivery, antepartum haemorrhage (APH), Rhesus and ABO immunisation, baby's sex, gestational age at birth, birth order, birth weight, jaundice, phototherapy, breast feeding (recorded at discharge), admission to neonatal unit and Apgar score.

Although the intention was to include all identified cases in the study, an *a priori *power calculation was performed for maternal age (the main factor of interest) based on an assumption that 10% of children without type 1 diabetes would have mothers who were 35 years or older at the time of their child's birth [18]. To have 90% power to detect a 10% difference in the proportions of children with older mothers, with a 5% two-sided significance level, assuming a proportion of discordant pairs of 15%, would require 90 cases and 270 controls [19]. If 25% of pairs were discordant, then 166 cases and 498 controls would be required to detect the same sized difference.

Unadjusted and adjusted odds ratios and their 95% confidence intervals were estimated using conditional logistic regression to evaluate associations between individual exposures and the risk of type 1 diabetes. Variables found to be significantly associated with type 1 diabetes in the univariate analyses were investigated further, by building multivariate models including possible confounding factors identified in the literature. Tests for interactions were performed to compare the risk factors for early (< 5 years) and later onset (5-14 years) type 1 diabetes, with the controls adopting the age of diagnosis of their matched case.

Continuous variables were grouped into categories for analysis. For variables with more than 10% missing values, an additional category for missing data was created. For other variables, missing values were excluded from the calculations. To confirm that no artificial group boundaries were generated by categorising the continuous variables, the above analyses were repeated retaining the continuous nature of the relevant variables. A *P*-value of < 0.05 was regarded as statistically significant. Statistical analyses were performed with SPSS (SPSS 15.0 for Windows, SPSS Inc., Chicago, Illinois, USA) and SAS (SAS for Windows, Release 9.1 SAS Institute Inc., Cary, NC, USA).

Ethical approval was granted by the North of Scotland Research Ethics Committee for all observational studies using routinely collected anonymised data from the AMND, provided permission was granted by the Steering Committee (Caldicott guardians) of the AMND. A request for access to data was approved by the Steering Committee on 5 March 2007.

## Results

There were 611 children diagnosed with type 1 diabetes in Grampian between 1984 and 2005. Fifteen children were born outside the period 1972 to 2002 and were excluded from the analysis. After further excluding children born outwith AMH (n = 226) and multiple births (n = 9), there were 361 children diagnosed with type 1 diabetes born between 1972 and 2002, inclusive. There were 139,480 singleton live births at AMH between 1972 and 2002, from which three randomly selected controls for each case, matched by the year of birth, were selected. A total of 361 children with type 1 diabetes and 1083 controls were included in this study.

The mothers of two cases and no controls had type 1 diabetes recorded during pregnancy. Paternal date of birth was not recorded for any participants. Unspecified viral infection was noted in only two control mothers and there were no recorded diagnoses of other bacterial disease. Rhesus immunisation was noted in only three control mothers and ABO immunisation in only one control mother. It was therefore not possible to explore these variables in more detail. Deprivation category, gestational age at delivery, birth weight and Apgar score at 1 and 5 minutes had less than 10% missing data, which were excluded from the analysis. An additional category was created for missing values for maternal BMI, maternal smoking, syntocinon, phototherapy, feeding at discharge and admission to neonatal unit, as they had a higher proportion of missing data.

The results of the conditional logistic regression analyses are shown in Tables 1 and 2. There was no significant association between maternal age at delivery and the risk of type 1 diabetes in the child (*P *= 0.87). Compared to a maternal age of 25 to 29 years at delivery, maternal age younger than 25 years had an OR of 1.00, 95% CI (0.75, 1.34), maternal age 30 to 34 years an OR of 1.12, 95% CI (0.81, 1.54) and maternal age of 35 and older an OR of 1.13, 95% CI (0.71, 1.79). A test for trend did not find an association between increasing maternal age and the risk of type 1 diabetes in the child (*P *= 0.48). After adjusting for amniocentesis, gestation at birth, birth weight, breast feeding, maternal deprivation, pre-eclampsia, birth order, mode of delivery and maternal smoking, there remained no significant association between maternal age and risk of type 1 diabetes (*P *= 0.82).

**Table 1:** Unadjusted and adjusted odds ratios for maternal risk factors and childhood type 1 diabetes

Maternal factor	T1D Cases n = 361	Controls n = 1083	Unadjusted			Adjusted*		
			
			OR	95% CI	*P*-value	OR	95% CI	*P*-value
	n (%)	n (%)						
**Maternal age (years)**					0.87			0.82
< 25	114 (31.6)	354 (32.7)	1.00	0.75, 1.34		1.09	0.78, 1.51	
25-29 (reference category)	126 (34.9)	391 (36.1)	1.00			1.00		
30-34	91 (25.2)	255 (23.5)	1.12	0.81, 1.54		1.05	0.74, 1.48	
≥ 35	30 (8.3)	83 (7.7)	1.13	0.71, 1.79		1.27	0.76, 2.13	
**Maternal BMI (kg/m^2 ^)**					0.14			0.55
< 25 (reference category)	167 (46.3)	547 (50.5)	1.00			1.00		
≥ 25	102 (28.3)	300 (27.7)	1.11	0.84, 1.47		1.14	0.85, 1.52	
Unknown	92 (25.5)	236 (21.8)	1.45	1.01, 2.10		1.26	0.75, 2.13	
**Previous abortions**					0.38			
No (reference category)	309 (85.6)	906 (83.7)						
Yes	52 (14.4)	177 (16.3)	0.86	0.61, 1.20				
**Maternal smoking**					**0.02‡**			0.14
No (reference category)	201 (55.7)	537 (49.6)	1.00			1.00		
Yes	84 (23.3)	330 (30.5)	0.61	0.43, 0.86		0.67	0.46, 0.99	
Unknown	76 (21.1)	216 (19.9)	0.74	0.45, 1.22		0.71	0.41, 1.26	
**Pre-eclampsia**					0.40			
No (reference category)	347 (96.1)	1029 (95.0)	1.00					
Yes	14 (3.9)	54 (5.0)	0.77	0.43, 1.40				
**Amniocentesis**					0.34			
No (reference category)	352 (97.5)	1045 (96.5)	1.00					
Yes	9 (2.5)	38 (3.5)	0.70	0.33, 1.47				
**Maternal deprivation category‡**			0.98	0.92, 1.04	0.51			
1 (least deprived)	102 (28.3)	320 (29.5)						
2	68 (18.8)	193 (17.8)						
3	50 (13.9)	112 (10.3)						
4	27 (7.5)	100 (9.2)						
5	23 (6.4)	94 (8.7)						
6	16 (4.4)	57 (5.3)						
7 (most deprived)	40 (11.1)	121 (11.2)						
Unknown	35 (9.7)	86 (7.9)						
**Syntocinon†**					0.32			
No (reference category)	168 (46.5)	478 (44.1)	1.00					
Yes	129 (35.7)	392 (36.2)	0.85	0.63, 1.17				
Unknown	64 (17.7)	213 (19.7)	0.67	0.40, 1.13				
**Mode of delivery**					0.54			
SVD (reference category)	241 (66.8)	753 (69.5)	1.00					
Assisted	67 (18.6)	187 (17.3)	1.12	0.82, 1.54				
Elective CS	24 (6.6)	53 (4.9)	1.42	0.86, 2.35				
Emergency CS	29 (8.0)	90 (8.3)	1.01	0.65, 1.58				
**APH**					0.79			
No (reference category)	327 (90.6)	986 (91.0)	1.00					
Yes	34 (9.4)	97 (9.0)	1.06	0.70, 1.60				
**Rhesus isoimmunisation**								
No (reference category)	361 (100.0)	1080 (99.7)						
Yes	0 (0.0)	3 (0.3)						
**ABO isoimmunisation**								
No (reference category)	361 (100.0)	1082 (99.9)						
Yes	0 (0.0)	1 (0.1)						

T1D, type 1 diabetes; OR, odds ratio; CI, confidence interval; BMI, body mass index; SVD, spontaneous vaginal delivery; CS, Caesarean section; APH, antepartum haemorrhage; kg, kilograms; m, metres. *Maternal age adjusted for amniocentesis, gestational age, birth weight, breast feeding, maternal deprivation, pre-eclampsia, birth order, mode of delivery and maternal smoking; BMI adjusted for gestation at the booking appointment; maternal smoking adjusted for maternal age, birth weight, breast feeding, maternal deprivation, pre-eclampsia, gestational age and mode of delivery. † Data on syntocinon collected from 1986 by AMND. ‡ P < 0.05. ‡ Risk of type 1 diabetes per unit increase in maternal deprivation.

**Table 2:** Unadjusted and adjusted odds ratios for neonatal risk factors for childhood type 1 diabetes

Neonatal factor	T1D Cases	Controls	Unadjusted			Adjusted*		
			
	n = 361	n = 1083	OR	95% CI	*P*-value	OR	95% CI	*P*-value
	n (%)	n (%)						
**Sex of baby**					1.00			
Male (reference category)	188 (52.1)	564 (52.1)	1.00					
Female	173 (47.9)	519 (47.9)	1.00	0.79, 1.27				
**Gestational age (weeks)**					0.41			
< 37	24 (6.6)	59 (5.4)	1.23	0.75, 2.02				
≥ 37 (reference category)	336 (93.1)	1023 (94.5)	1.00					
Unknown	1 (0.3)	1 (0.1)						
**Birth Order**					0.84			
1 (reference category)	181 (50.1)	525 (48.5)	1.00					
2	117 (32.4)	357 (33.0)	0.95	0.74, 1.24				
≥ 3	63 (17.5)	201 (18.6)	0.91	0.65, 1.27				
**Birth weight (kg)**					0.50			0.22
<2.5	17 (4.7)	61 (5.6)	0.82	0.47, 1.44		0.66	0.34, 1.28	
≥ 2.5 (reference category)	344 (95.3)	1021 (94.3)	1.00			1.00		
Unknown	0 (0.0)	1 (0.1)						
**Jaundice**					0.75			
No (reference category)	338 (93.6)	1018 (94.0)	1.00					
Yes	23 (6.4)	65 (6.0)	1.10	0.60, 2.04				
**Phototherapy†**					0.42			
No (reference category)	41 (11.4)	101 (9.3)	1.00					
Yes	9 (2.5)	30 (2.8)	0.72	0.31, 1.68				
Unknown	311 (86.1)	952 (87.9)	0.74	0.46, 1.18				
**Breast feeding**					0.66			0.26
No (reference category)	23 (6.4)	80 (7.4)	1.00					
Yes	32 (8.9)	90 (8.3)	1.27	0.67, 2.42		1.62	0.77, 3.44	
Unknown or mixed feeding‡	306 (84.8)	913 (84.3)	1.49	0.56, 3.92		2.24	0.78, 6.43	
**Admitted to neonatal unit†**					0.27			
No (reference category)	90 (24.9)	276 (25.5)	1.00					
Yes	12 (3.3)	38 (3.5)	0.98	0.48, 2.00				
Unknown	259 (71.7)	769 (71.0)	3.47	0.76, 15.91				
**Apgar score at 1 min**					0.39			
1 to 7	89 (24.7)	245 (22.6)	1.13	0.85, 1.49				
8 to 10 (reference category)	271 (75.1)	836 (77.2)	1.00					
Unknown	1 (0.3)	2 (0.2)						
**Apgar score at 5 min**					0.45			
1 to 7	14 (3.9)	33 (3.0)	1.28	0.68, 2.40				
8 to 10 (reference category)	346 (95.8)	1049 (96.9)	1.00					
Unknown	1 (0.3)	1 (0.1)						

T1D, type 1 diabetes; OR, odds ratio; CI, confidence interval; kg, kilograms. *Birth weight adjusted for gestation; breast feeding adjusted for deprivation. †Data on phototherapy and admission to neonatal unit recorded from 1992 by AMND. ‡This category includes one case and 3 controls who were breast and formula fed.

A significantly decreased risk of type 1 diabetes was observed in children whose mothers smoked at booking appointment compared to those whose mothers did not, with an OR of 0.61, 95% CI (0.43, 0.86). After adjusting for maternal age, birth weight, breast feeding, maternal deprivation, pre-eclampsia, gestational age and mode of delivery, the overall association between maternal smoking and type 1 diabetes was no longer significant (*P *= 0.14). However, one of the adjusted ORs from this analysis suggested a reduced risk in children whose mothers smoked, compared to children whose mothers did not smoke (adjusted OR of 0.67, 95% CI (0.46, 0.99)). To investigate this possible association further, a sensitivity analysis was performed firstly by excluding all those with an unknown maternal smoking status, then by assuming that all unknowns smoked, and finally by assuming that all unknowns did not smoke. This sensitivity analysis produced similar adjusted ORs and 95% CIs, with adjusted *P *= 0.10, *P *= 0.05, and *P *= 0.10, respectively.

There was no evidence of statistically significant associations between type 1 diabetes in the child and any of the other risk factors studied: maternal BMI, previous abortions, pre-eclampsia, amniocentesis, maternal deprivation, syntocinon, mode of delivery, APH, baby's sex, gestational age at birth, birth order, birth weight, jaundice, phototherapy, breast feeding, admission to neonatal unit and Apgar score (*P *> 0.05).

There were 86 children diagnosed with type 1 diabetes under 5 years of age (early onset) and 275 diagnosed between 5 and 14 years of age (later onset). There was a significant interaction between age of diagnosis and whether the mother had previous abortions (*P *= 0.03). The mothers of cases diagnosed before age 5 were more likely to have had previous abortions than the mothers of their controls (27% versus 20%), while the mothers of cases diagnosed later were less likely to have had previous abortions than the mothers of their controls (11% versus 15%). None of the other risk factors studied showed significant interactions with age of diagnosis (*P *> 0.05; data available upon request).

Retaining the continuous nature of maternal age, maternal BMI, birth weight and gestational age at booking appointment and at birth produced similar results to the above analysis (data available upon request).

## Discussion

This study found some evidence of a reduced risk of children developing type 1 diabetes under 15 years of age if their mothers smoked, compared to children whose mothers did not smoke, with an adjusted OR of 0.67, 95% CI (0.46, 0.99). However, this should be interpreted cautiously as smoking status was self-reported and there was a large amount of missing data (20%). After adjusting for possible confounders, this study found no evidence that other maternal and neonatal factors studied had a significant association with the subsequent development of type 1 diabetes under 15 years of age.

A major strength of this study was the source of the participants and data. The two data sources have been shown to be complete and valid [15,20]. In contrast with most retrospective studies, all pregnancy events were recorded by AMND staff at the time of the event, without any possibility of being influenced by knowing if children went on to develop type 1 diabetes. In addition, the study did not rely on mothers' memory of past events, thus eliminating any potential for recall bias. The controls were selected from the same population as the cases and the exposure data for cases and controls were collected from the same source. A further strength was the large range of maternal and neonatal factors recorded, which allowed for appropriate investigation of confounding and adjustment of the analyses. Over the period of the study, there have been changes both in the incidence of type 1 diabetes and in environmental exposures. To minimise the potential confounding effect of factors that changed over time, cases and controls were individually matched on the year of birth.

The study, however, has several limitations. The size of the study could mean we had limited power to detect the small effects currently being described in meta-analyses of perinatal risk factors [11-13]. It was not possible to rule out the effect of other potential confounders, such as paternal age, family history of diabetes and unknown confounders, which were not recorded in the AMND. A further limitation was that three of the variables (syntocinon, phototherapy and admission to neonatal unit) were not recorded throughout the entire study period and several of the variables had missing values, which could bias the results.

In addition, the AMND does not collect information on home births or on births in rural maternity units, unless they are subsequently transferred to AMH due to complications. Of the 241 excluded cases, 15 (6%) were born outside 1972 to 2002. No information was available about whether the remaining excluded cases were home delivered, delivered in another Grampian hospital or inward migrants who were born outside Aberdeen. However, there were relatively few home births in Aberdeen and Aberdeenshire, about 7 per 1,000 births, in 1998 [21]. The proportion of live births in Grampian recorded in other maternity units in 2005 was 22%[22].

It is possible that some children selected as controls will have migrated out of the Grampian area and subsequently been diagnosed with type 1 diabetes. If migration rates were related to the risk factors studied, this may lead to bias. However, there is evidence of geographic stability among Aberdonians [23]. Children diagnosed with type 1 diabetes before the SSG register was started in 1984 would not have been identified and may have been selected as a control. In addition, controls born after 1990 and not identified as having type 1 diabetes up to the end of 2005 may subsequently develop type 1 diabetes by the age of 15 years. However, misclassification as a result of the above scenarios would probably only equate to a few additional cases.

Univariate analysis revealed a significant decreased risk in children whose mothers smoked during early pregnancy compared to those who did not, with limited evidence supporting this hypothesis after adjusting for potential confounders. The finding of a protective effect of smoking is consistent with three other case-control studies [24-26]. However, one of these studies did not adjust for social class [24] and the other two studies were based on questionnaire data [25,26]. The majority of studies have not observed a significant association between maternal smoking and risk of type 1 diabetes, including two cohort studies from the UK [9,10]. However, self-reported data on maternal smoking status were collected at the mother's booking appointment, which may be unreliable and it is unknown how many mothers continued to smoke during their pregnancy or the number of cigarettes smoked per day. In addition, it was not possible to adjust for paternal smoking, which has been found to be significantly associated in one study [10]. Also, given the number of statistical tests performed, this significant result could be due to chance and a Type 1 error may not be ruled out.

No association was found between maternal age at birth and the risk of type 1 diabetes in the child, after adjusting for possible confounders. This finding is consistent with some studies [9,10]. However, an increased risk in children born to older mothers has recently been reported [11]. The pooled analysis of 30 observational studies demonstrated that there was, on average, a 5% (95% CI 2, 9) increase in childhood type 1 diabetes odds per 5-year increase in maternal age (*p *= 0.006) [11]. Our study may not have had sufficient power to detect such a small effect. In addition, we were unable to rule out the effect of other possible confounders such as paternal age and maternal diabetes.

The findings of this study could not confirm the increased risk found in a meta-analysis, which reported a small, but significant, adjusted OR of 1.23, 95% CI (1.15, 1.32, *P *< 0.001) for Caesarean delivery [12]. Again, a lack of power could explain this difference in findings.

No association was found between birth weight and risk of type 1 diabetes, which is in agreement with some case-control studies [5,14,27,28]. However, a recent meta-analysis concluded that children who are heavier at birth have a significant and consistent, but relatively small increase in risk of type 1 diabetes. The authors reported that children with birth weight from 3.5 to 4 kg had an increased risk of diabetes of 6% (OR 1.06, 95% CI (1.01, 1.11); *p *= 0.02) and children with birth weight over 4 kg had an increased risk of 10% (OR 1.10, 95% CI (1.04, 1.19); *p *= 0.003), compared with children weighing 3.0 to 3.5 kg at birth [13].

The protective effect of breast feeding reported by systematic reviews [29-31] was not confirmed in this study. However, data on infant feeding were collected at the mothers' discharge from hospital, and it is unknown how many mothers continued to breast feed after this time. The finding of this study is, however, similar to that reported by other UK studies [9,14,32].

There was no evidence of an association between maternal BMI and the risk of type 1 diabetes in the child, consistent with previous studies [14,24,33,34] with the exception of one [35], which observed an increased risk with excessive weight gain during pregnancy. The ideal time to record the BMI of a pregnant woman is before she has started gaining weight due to gestation. In this study, BMI was recorded at the first booking appointment (by 16 weeks gestation), before any real impact of gestational weight gain; however, values recorded remain an approximation of the pre-pregnancy weight. Also, not all women had their booking appointment by 16 weeks gestation. To adjust for this, gestation at booking appointment was included in the conditional logistic regression model when deriving adjusted ORs for BMI. It was not possible to investigate weight gain during pregnancy in this study.

A significant interaction was found between age of diagnosis and whether the mother had previous abortions. There were no other differences in risk factors between early and later onset type 1 diabetes, which is consistent with some studies [6,14,35]. However, two studies have observed that a first pregnancy was a more important risk factor for early than for later onset type 1 diabetes [5,8]. Dahlquist and colleagues [36] reported that for onset before 10 years of age, there was a significant association with birth weight, while cases with onset at the age of 10-29 showed no significant trend.

## Conclusions

This study found limited evidence of a reduced risk of developing type 1 diabetes under the age of 15 years in children whose mothers smoked, compared to those whose mothers did not. No evidence was found of a significant association between a range of other maternal and neonatal factors and the subsequent development of childhood type 1 diabetes. Systematic reviews and meta-analyses have been conducted on maternal age [11], Caesarean section [12], birth weight [13]and infant nutrition [29-31] and the risk of childhood type 1 diabetes. Further studies would be useful in synthesising the available evidence for other maternal and neonatal factors.

## Competing interests

The authors declare that they have no competing interests.

## Authors' contributions

LR supervised the data collection, carried out the majority of the data analysis and drafted the manuscript. KH performed the sample size calculation, carried out the matching of cases to controls, and performed some of the data analysis. Both authors participated in the study design and coordination, contributed to the interpretation and approved the final manuscript.

## Pre-publication history

The pre-publication history for this paper can be accessed here:

http://www.biomedcentral.com/1471-2458/10/281/prepub
